# Total Elbow Arthroplasty: Tips and Tricks in the Setting of Arthritis and Fracture Care

**DOI:** 10.1002/atn2.70209

**Published:** 2026-08-07

**Authors:** Benjamin Kerzner, Kyleen Jan, Alexander L. Hornung, John F. Hoy, Xavier C. Simcock

**Affiliations:** ^1^ Department of Orthopaedic Surgery Rush University Medical Center Chicago Illinois U.S.A.

## Abstract

The aim of this article is to illustrate our technique for total elbow arthroplasty, acknowledging the key technological advancements and evidence‐based techniques that have emerged in the last 2 decades. In this technical note, we describe our pearls for a reproducible and reliable total elbow arthroplasty procedure. We also provide evidence‐based recommendations on implant selection, surgical approach, management of the triceps, and situations where total elbow arthroplasty is more beneficial over open reduction internal fixation for distal humerus fractures.

## Graphical abstract

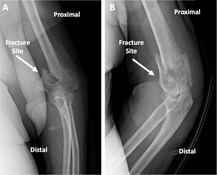

**VIDEO 1:** In this technical note, we describe our tips and tricks for a reliable and reproducible left elbow total elbow arthroplasty procedure with evidence‐based pearls in the management of both arthritis and fracture care. Video content can be viewed at https://doi.org/10.1002/atn2.70209.

The use of total elbow arthroplasty (TEA) as a treatment option for elbow arthritis is increasing worldwide. The primary goal of TEA is to alleviate pain and restore an acceptable range of motion to the elbow joint. The history of TEA originates in the 1940s through the use of hemiarthroplasties or custom distal humerus or proximal ulna articular‐bearing material components.^1^ Although “modern” TEA is credited to Dee in 1972, numerous design iterations such as the “saddle,” a “true” olecranon resurfacing,” developed by Morrey in the late 1960s, resulted in the evolution of the progression from unlinked (unconstrained) to linked (semiconstrained) and finally the convertible prosthesis. The convertible prosthesis allows for conversion of an unlinked prosthesis to a linked prosthesis if the implant is unstable intraoperatively or postoperatively, ultimately widening the indications for TEA.^2^ Initially, rheumatoid arthritis (RA) was the primary indication for TEA; over time, its indications have expanded to encompass unreconstructable distal humerus fracture in the elderly (Figure 1A,B), primary and secondary osteoarthritis, nonunion, and post‐tumor resection.^3^

**FIGURE 1:**
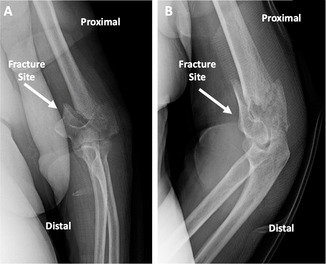
Preoperative radiographs. Anteroposterior (A) and lateral (B) radiographs showing a left elbow displaced, comminuted, and intra‐articular distal humerus fracture. There is no evidence of skin tenting or open fracture.

TEA is not without its pitfalls as complications such as infection, aseptic loosening, polyethylene wear, periprosthetic fracture, neuropathy, and triceps insufficiency initially plagued patients.^4^ Continued scrutiny of operative criteria, advancements in implant design, consideration of patient pathology, and evolution of surgical techniques substantially decreased complication rates from 50% to ∼10% in recent case series.^5^, ^6^, ^7^, ^8^, ^9^, ^10^ Moreover, studies focusing on TEA in patients with RA have reported promising outcomes with recent research showing 97% survival at 5 years and 85% at 10 years.^11^, ^12^

TEA is a valuable surgical intervention that offers improved outcomes for various elbow conditions. The significance of different techniques lies in their ability to ensure proper alignment, restore function, and alleviate pain, ultimately enhancing the quality of life for patients undergoing TEA. Here, we describe our tips and tricks for TEA in the setting of arthritis and fracture care.

## SURGICAL TECHNIQUE

### Patient Positioning and Anesthesia

**FIGURE 2:**
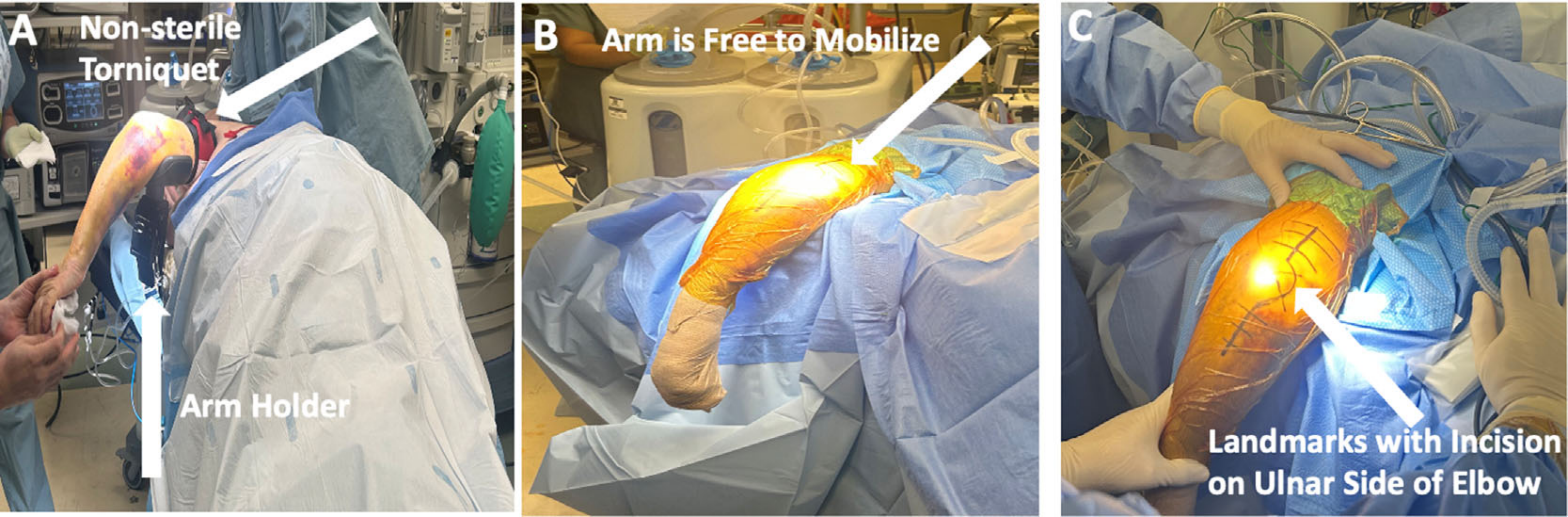
Positioning and landmarks. The left elbow is placed in an arm holder coming from the contralateral side of the bed, oriented so to rest on the anterior surface of the middle third of the humerus during the surgical procedure (A). A nonsterile tourniquet is applied once the patient is appropriately positioned in the lateral decubitus. An axillary roll is placed under the nonoperative arm, and the operative extremity is then prepped and draped in the usual sterile fashion. Ioban is placed sterilely over the posterior arm and elbow (B). The medial and lateral epicondyles are marked as well as the olecranon process. Radial and ulnar sides should be clearly indicated with a sterile marker on the operative extremity to ensure proper anatomic orientation is maintained throughout the entirety of the case (C).

**FIGURE 3:**
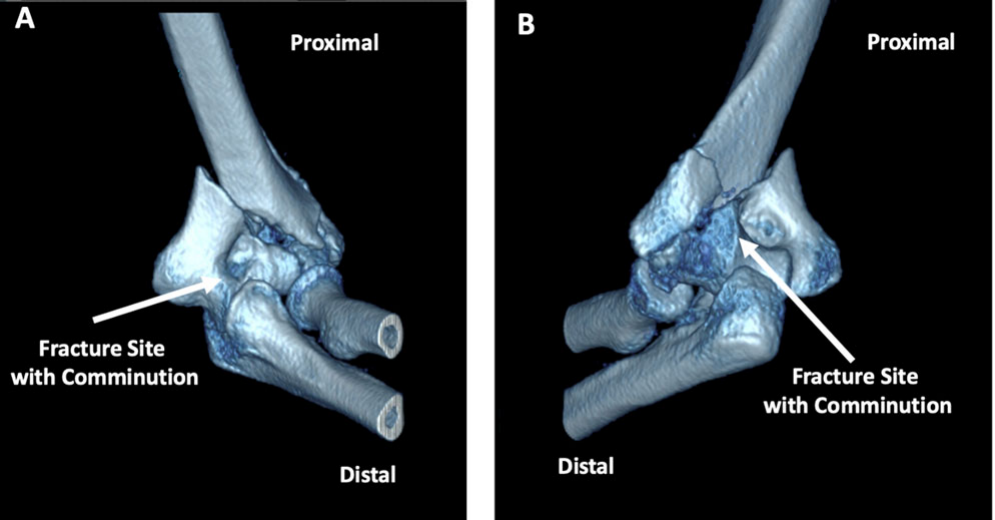
Computed tomography. Three‐dimensional reconstruction computed tomography scan of the left elbow (A,B) showing significant comminution of the articular block of the distal humerus as well as intra‐articular involvement.

After administration of a regional supraclavicular block preoperatively for pain control, the patient is brought to the operating room. After anesthesia is induced, the patient is placed in the lateral decubitus position with use of a beanbag. An arm holder coming from the contralateral side of the injury when the patient is supine is oriented so to rest on the anterior surface of the middle third of the humerus during the surgical procedure (Figure 2A, Video 1). A nonsterile tourniquet is applied once the patient is appropriately positioned in the lateral decubitus. A sterile tourniquet can also be used if the planned procedure extends more proximally along the humerus. An axillary roll is placed under the nonoperative arm, and the operative extremity is then prepped and draped in the usual sterile fashion. Ioban is placed sterilely over the posterior arm and elbow (Figure 2B). The tourniquet is then inflated to 250 mm Hg. In the setting of a fracture, palpable landmarks may be altered, and it is critical to have all preoperative imaging in the operating room including preoperative computed tomography (Figure 3A,B).

### Surgical Dissection and Exposure

Prior to surgical incision, the medial and lateral epicondyles are marked as well as the olecranon process. Radial and ulnar sides should be clearly indicated with a sterile marker on the operative extremity to ensure proper anatomic orientation is maintained throughout the entirety of the case (Figure 2C). A standard posterior incision is used, starting at about 6 to 7 cm proximal to the olecranon and extending in a curvilinear fashion around the ulnar aspect of the olecranon and 4 to 5 cm distal to the joint. The incision is begun first with a 10 blade and followed by use of Metzenbaum scissors to create large subcutaneous flaps. Dissection should go as deep to the triceps tendon, but the paratenon should be kept intact. Care on the ulnar side should be kept superficial to the fascia of the ulnar nerve.

**FIGURE 4:**
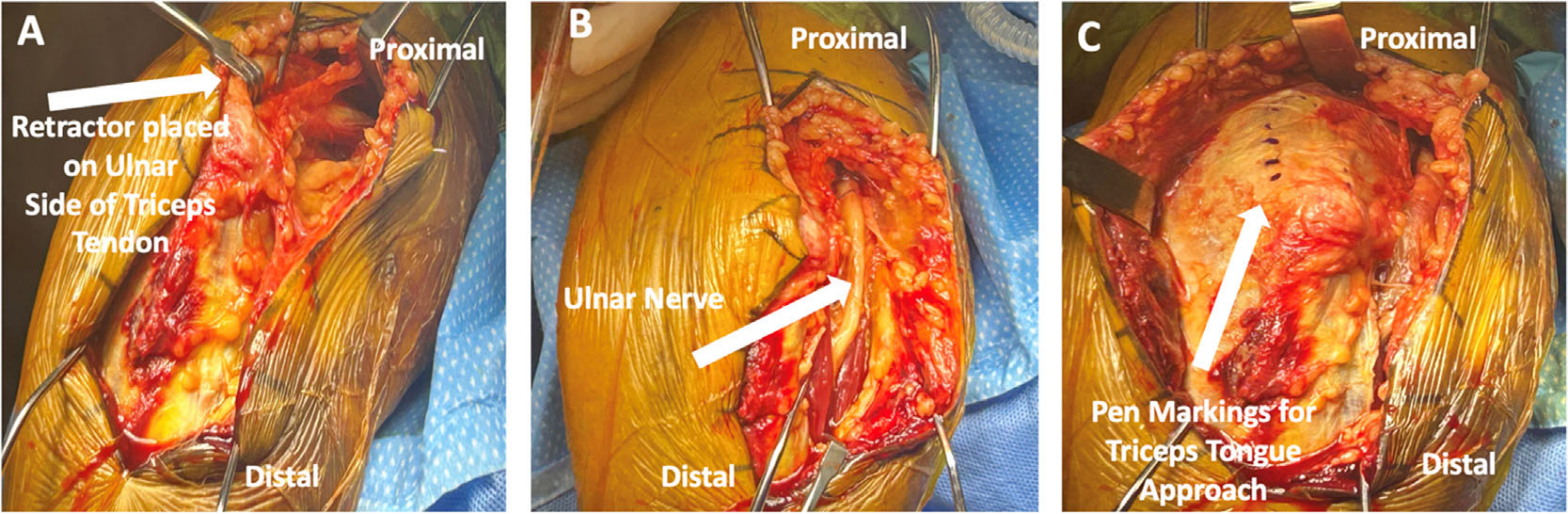
Ulnar nerve and triceps management. A Senn retractor is placed on the left elbow triceps tendon near its insertion on the olecranon and pulled radially to help expose the nerve (A). The ulnar nerve should first be decompressed proximally and then traced distally all the way to the FCU. Once the FCU fascia is split, the ulnar nerve will now be free to fall anteriorly away from the elbow joint (B). The triceps tendon should be incised longitudinally with a sharp knife in a 10 × 2 cm size and reflected from proximal to distal. Bovie must not be used here as it will devitalize the tendon. Once reflected distally, the triceps tendon can be sutured to the fascia on the olecranon using Vicryl suture (C). (FCU, flexor carpi ulnaris.)

Two Gelpi retractors are then used, and attention is next turned to identify the ulnar nerve from the ulnar aspect of the triceps. A Senn retractor is placed on the triceps tendon near its insertion on the olecranon and pulled radially to help expose the nerve (Figure 4A). The ulnar nerve should first be decompressed proximally and then traced distally all the way to the flexor carpi ulnaris. Once the flexor carpi ulnaris fascia is split, the ulnar nerve will now be free to fall anteriorly away from the elbow joint (Figure 4B). Complete transposition will be completed at the end of the case. Even though the nerve is now decompressed, the ulnar nerve should be checked multiple times throughout the whole case to ensure no accidental injury.

The workhorse of this approach is the triceps tongue approach (Figure 4C). The triceps tendon should be incised longitudinally with a sharp knife in a 10 × 2 cm size and reflected from proximal to distal maintaining its insertion on the olecranon. Bovie must not be used here as it will devitalize the tendon. Once reflected distally, the triceps tendon can be sutured to the fascia on the olecranon using Vicryl suture. Ulnar and radial sides of the olecranon process must be exposed in order to reflect the proximal ulna out of the joint. The coronoid should be visible at this point in the case. Again, the ulnar nerve should frequently be visualized and palpated during this step.

**FIGURE 5:**
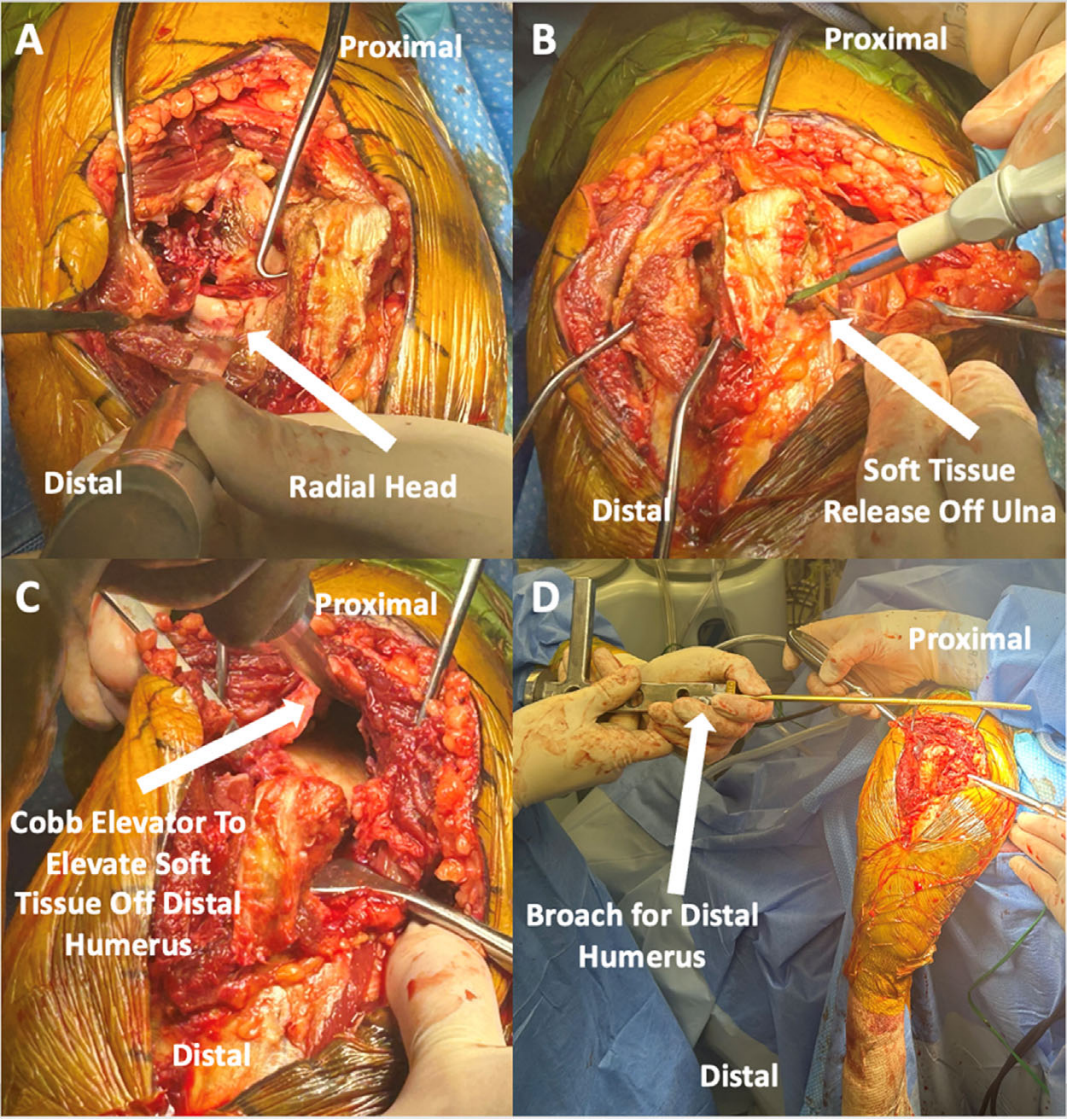
Humeral bony preparation. The left elbow radial head should be cut with an oscillating saw proximal to the level of the proximal radioulnar articulation (A). The lateral ulnar collateral ligament and the medial collateral ligament need to be released to ultimately raise the distal humerus out of the wound, and care should be taken to stay on the bone during soft tissue release to avoid accidental iatrogenic nerve injury (B). A Cobb elevator can be used to gently release soft tissue off the humerus as any soft tissue left could impede the anterior phalange of the humeral prosthesis (C). Next, a broach should be used for the distal humerus until an appropriate fit and then the humeral component can be trialed (D).

Frequently, the radial head is removed during TEA, and thus, subperiosteal dissection should be continued to the proximal radioulnar joint. The radial head should be cut with an oscillating saw proximal to the level of the proximal radioulnar articulation (Figure 5A) with Hohmann retractors carefully placed to protect the PIN anteriorly.

### Ulnar and Humeral Preparation

Attention is then turned to soft tissue management of the distal humerus. It is critical that the entire humeral epicondylar axis is visualized in order to gauge correct rotation. The lateral ulnar collateral ligament as well as the medial collateral ligament origins need to be released to ultimately raise the distal humerus out of the wound (Figure 5B). Care should be taken to stay on the bone during soft tissue release to avoid accidental iatrogenic nerve injury. A Cobb elevator can be used to gently release soft tissue off the anterior humerus as any soft tissue left could impede the anterior phalange of the humeral prosthesis (Figure 5C). The extrafossa humeral guide (Discovery Elbow System; Enovis; Wilmington, DE) should be lined up to the humeral epicondylar axis with the guide. Do not line the guide up distal to the epicondyles as this will result in the prosthesis being too tight in extension. In the setting of arthritis, use a Bovie to mark the most proximal extent of where the prosthesis will sit (where the proximal cut will be) as well as the ulnar/radial sides to determine the width of the box that will be cut off the distal humerus. With the anterior soft tissue protected with Chandler retractors, the box cut is made with a sagittal saw. A burr will then be used to open up the canal at the inflection point where the anterior humerus slopes to meet the posterior aspect of the humerus. Next, broach until an appropriate fit and trial the humeral component (Figure 5D).

**FIGURE 6:**
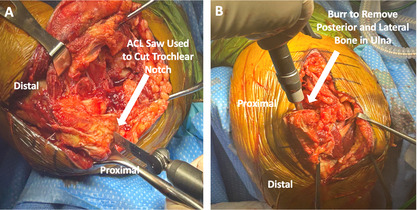
Ulnar bony preparation. An ACL saw can be used to cut subchondral bone to the trochlear notch of the olecranon of the left elbow. This step is crucial to ultimately be able to posteriorize the prosthesis (A). A high‐speed burr is then used to burr out the posterior and lateral bone which otherwise can prevent the correct positioning of the ulnar component (B). The burr also assists in opening up the canal, and then, a canal finder with a modular T‐handle is applied. (ACL, anterior cruciate ligament.)

On the ulnar side, an anterior cruciate ligament saw can be used to cut subchondral bone to the trochlear notch of the olecranon. This step is crucial to ultimately be able to posteriorize the prosthesis (Figure 6A). Next, a high‐speed burr is used to burr out the posterior and lateral bone which otherwise can prevent the correct positioning of the ulnar component (Figure 6B). The burr also assists in opening up the canal and then a canal finder with a modular T‐handle is applied. When the surgeon is burring or broaching, they must hold the ulna between their index finger and thumb to control movement in the coronal plane and avoid perforation of the anterior cortex. The ulnar trial component (Discovery Elbow System; Enovis; Wilmington, DE) can then be seated in place.

**FIGURE 7:**
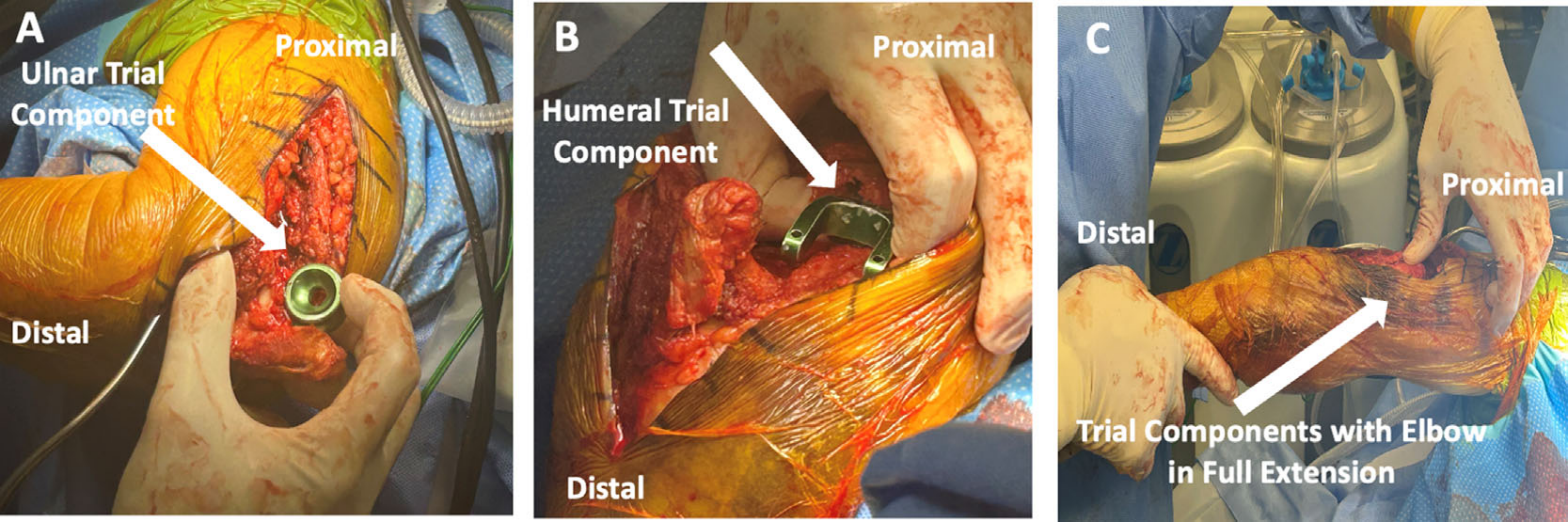
Trialing implants. The left elbow ulnar (A) and humeral (B) trial components should be placed in respective intramedullary positions, and the elbow should be brought to achieve extension of the arm (C). It is not necessary to force the elbow to full extension if it cannot be achieved at this point, as you do not want to put more stress on the implant.

With both the ulnar (Figure 7A) and humeral (Figure 7B) trial components in place, the arm should be brought to achieve extension of the arm (Figure 7C). Do not force the elbow to full extension if it cannot be achieved at this point, as you do not want to put more stress on the implant. Rotation should also be assessed here and want about 10° of internal rotation. Finally, check the anterior phalange and ensure no soft tissue is impinging.

### Final Implant Placement

**FIGURE 8:**
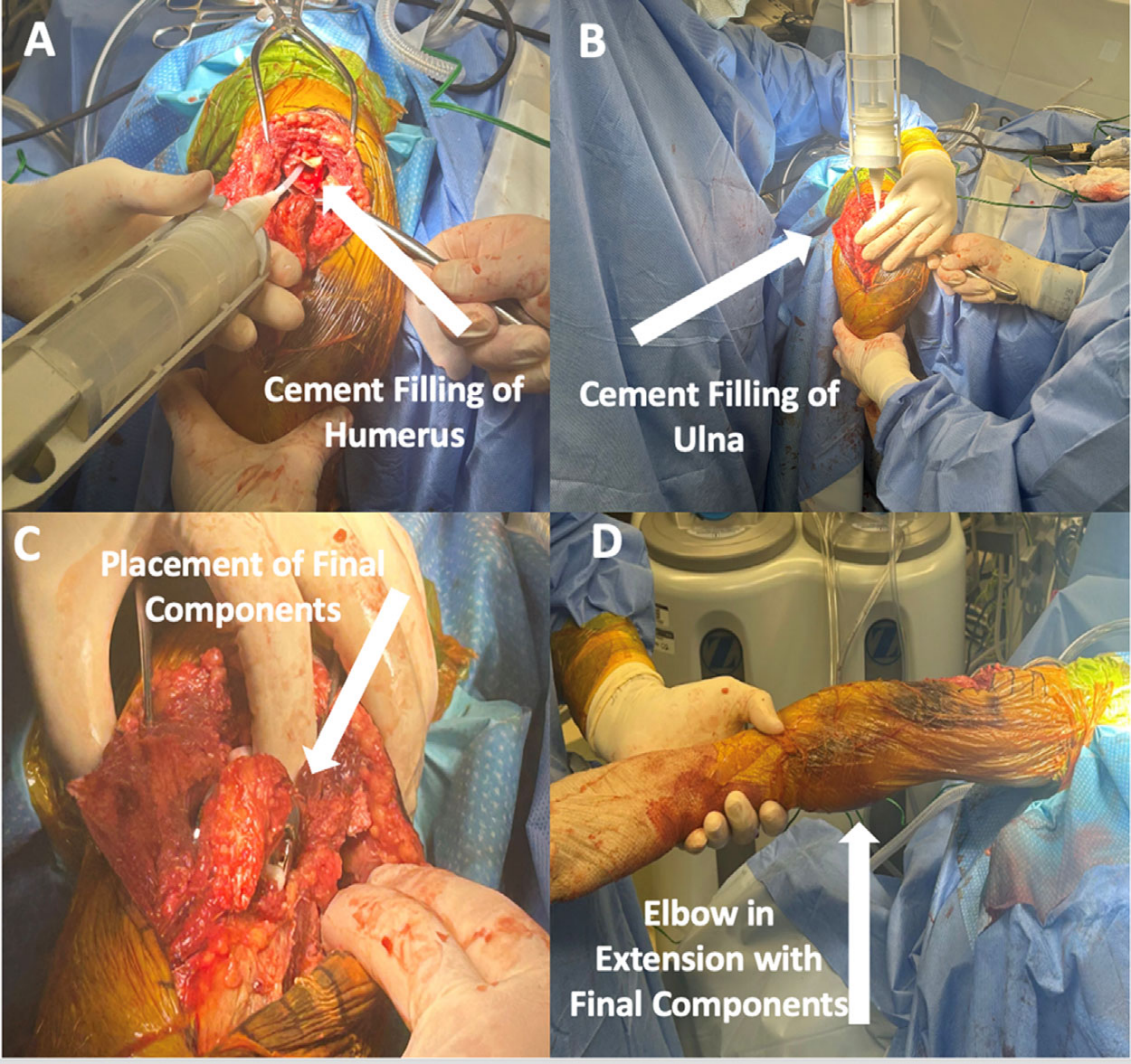
Cementing and placement of final components. The left elbow is hyperflexed, and valgus stress is placed to deliver the joint up out of the wound and fill the canals with cement (A,B). Once the complete prosthesis is placed in flexion (C), the arm should be brought into extension and held until the cement has dried (D). During this time, ensure that there is no cement on the bearings, condyles, and poly.

Once the final humeral and ulna sizes have been determined, all the components (Discovery Elbow System; Enovis; Wilmington, DE) should be assembled on the back sterile table. At this point, 2 strips of Surgicel are placed in the canals as a cement plug. Ideally, there needs to be about 2 cm of cement mantle in the canal for each component. Hyperflex the elbow and place valgus stress to deliver the joint up out of the wound and fill the canals with cement (Figure 8A,B). The surgeon's thumb can be used to pack the canal. Once the complete prosthesis (Discovery Elbow System; Enovis; Wilmington, DE) is placed in flexion (Figure 8C), take the arm into extension and hold the arm in extension until the cement has dried (Figure 8D). During this time, ensure that there is no cement on the bearings, condyles, and poly.

### Closure

**FIGURE 9:**
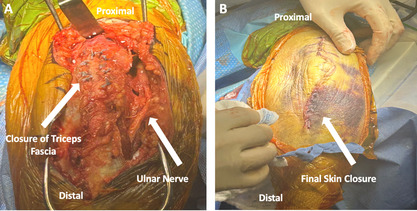
Closure. The left elbow triceps fascia is closed with #2 FiberWire (Arthrex, Naples, FL) (A), vancomycin powder is applied to the wound topically, and then, the subcutaneous layers are closed with 2‐0 Vicryl followed by 4‐0 Nylon (B). A splint is placed in 60° of flexion to protect the triceps repair.

**FIGURE 10:**
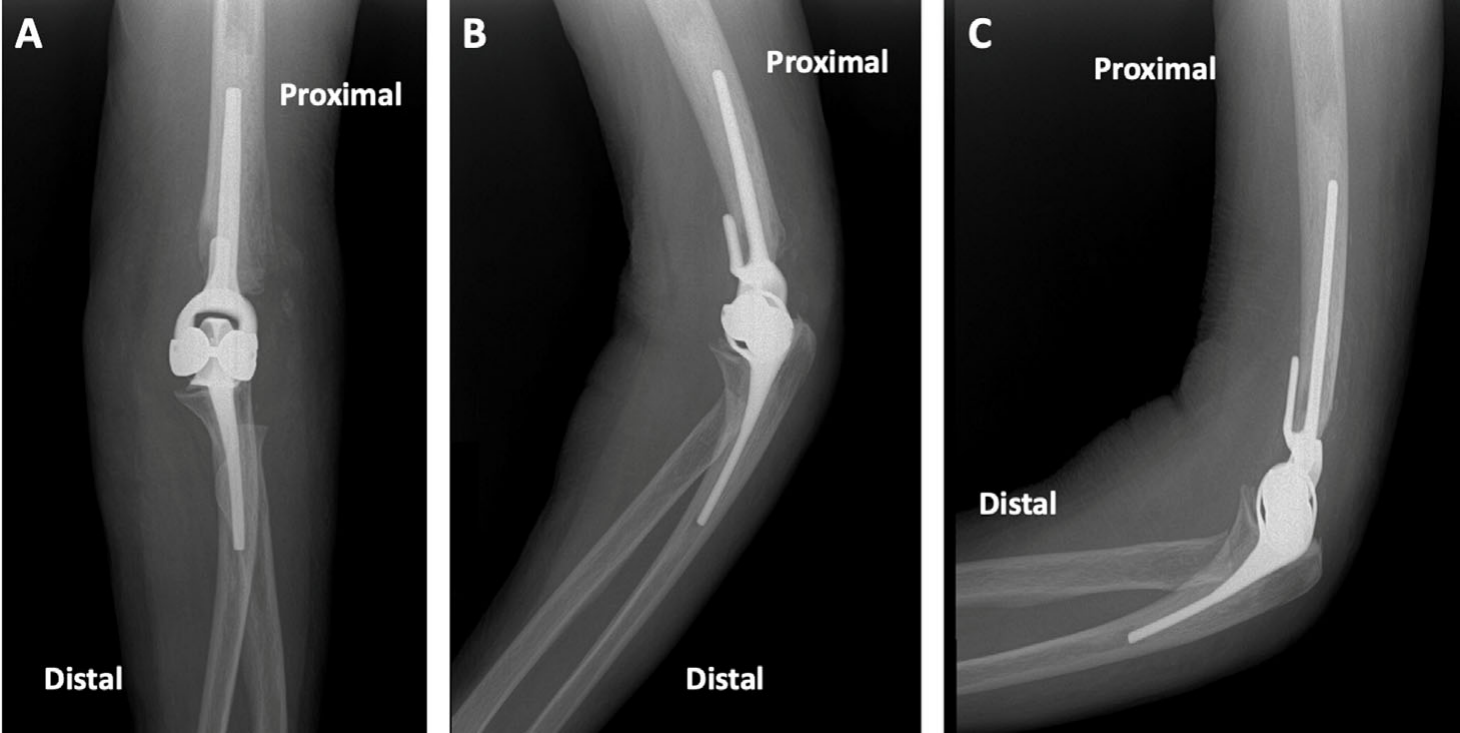
Final postoperative radiographs. Anteroposterior (A), oblique (B), and lateral (C) radiographs showing a left elbow total elbow arthroplasty semiconstrained components in proper positioning.

The tourniquet is then released and electrocautery is used to coagulate and smaller bleeding vessels. The elbow is taken through the full range of motion at this time and then copiously irrigated with saline. The triceps fascia is closed with #2 FiberWire (Arthrex, Naples, FL) (Figure 9A), vancomycin powder is applied to the wound, and then, the subcutaneous layers are closed with 2‐0 Vicryl followed by 4‐0 Nylon (Figure 9B). A splint is placed in 60° of flexion to protect the triceps repair. Postoperative radiographs are obtained to ensure proper component positioning (Figure 10A‐C).

### Rehabilitation

The patient is seen in the office for a skin evaluation at 10 to 14 days. Sutures are removed and the patient is placed in an elbow Orthoplast at 60° with a posterior cutout for the olecranon to avoid any skin breakdown. Patients begin with passive range of motion and active flexion of the elbow from 2 to 6 weeks postoperatively. Active extension is avoided to protect the triceps tongue repair. At 6 weeks, the patient can wean from the Orthoplast and begin active and passive range of motion. Once the range of motion goals are met, strengthening can begin with a weight limit of 5 or 10 lbs based on the surgeons’ discretion.

## DISCUSSION

The aim of this article is to illustrate our technique for TEA, acknowledging the key technological advancements and evidence‐based techniques that have emerged in the last 2 decades. Table 1 highlights the pearls and pitfalls from our technique for TEA. We highlight implant design, management of the triceps intraoperatively, considerations for TEA for a variety of pathologies, and the use of TEA over open reduction internal fixation (ORIF) in the setting of fracture.

**TABLE 1:** Pearls and Pitfalls

Pearls
• During initial dissection, large subcutaneous flaps should be elevated on either side of the triceps tendon and the paratenon should not be violated. This is crucial for the ultimate proper repair and closure of the triceps at the end of the case
• Ulnar nerve decompression is most easily achieved by starting proximally and then tracing the nerve distally all the way to the FCU. Once the FCU fascia is split, the ulnar nerve will now be free to fall anteriorly away from the elbow joint
• Both the LUCL and the MCL origins need to be released to ultimately raise the distal humerus out of the wound for bony preparation
• While trialing the components, do not force the elbow into full extension if it cannot be achieved gently as this will place unintended stress on the implant
• Surgicel should be placed in the intramedullary canal of both the humerus and ulna to act as a cement plug prior to delivering the cement

Pitfalls
• Using a Bovie, instead of a knife, during the triceps tongue approach, will result in devitalization of the triceps tendon
• Do not line the extrafossa humeral guide up distal to the epicondyles as this will result in the prosthesis being too tight in extension
• Failure to perform the osteotomy on the ulna at the correct level will result in the ulnar component not being able to be placed in the needed posterior position
• Not placing the patient in a splint at about 60° of flexion at the end of the case can compromise the triceps repair

FCU, flexor carpi ulnaris; LUCL, lateral ulnar collateral ligament; MCL, medial collateral ligament.

### Implant Design: Nonconstrained Versus Semiconstrained

Generally, 2 categories of implants exist today: unlinked and linked. Unlinked (also known as nonconstrained) implants consist of separate humeral and ulnar metal components which articulate solely via a polyethylene component and rely on bony support and collateral ligaments rather than a bushing‐axle linkage and pin for stability.^13^ This design decreases mechanical loosening from bone but carries a higher risk of subluxation/dislocation, particularly in rheumatoid populations.^14^, ^15^

On the contrary, linked designs describe stemmed implants with semiconstrained (also referred to as “sloppy hinge”) articulation. The humeral and ulnar components are linked with a bushing and axle configuration but still allow for movement in the coronal plane (varus‐valgus motion) due to a circular polyethylene ring between the metal components. These implants have greater stability compared with unlinked prostheses. However, most designs still only allow for a small area of contact between the humeral and ulnar components, leading to higher concentrated contact stresses on the polyethylene even with small varus‐valgus laxity.^13^ Long‐term outcomes still remain inferior to total hip or knee arthroplasty with 10‐year failure rates ranging from 14% to 35%.^16^, ^17^ As such, many surgeons limit TEA patients to 5‐ or 10‐pound weight‐bearing restrictions to minimize aseptic loosening and wear‐related complications.^18^ This presents a conundrum as the indications for TEA have expanded and the demographic of patients undergoing TEA has increasing activity demands.^18^ Rates of patient adherence to these restrictions and whether the restrictions truly influence failure rates remain unknown. As such, there is a need for continued innovation and research on polyethylene wear, osteolysis, and patient optimization in the TEA population.^16^, ^19^, ^20^, ^21^

### Surgical Approach and Triceps Management

The choice of surgical approach for TEA can significantly impact intraoperative visualization and subsequent outcomes.^22^, ^23^ During surgical exposure, care should be taken to account for the proximity of neurovascular structures, thin soft tissue envelope, potential prior hardware, existing bone loss, and potential injury to the triceps extensor mechanism.^24^, ^25^ Generally, employed approaches include the triceps‐splitting, triceps‐reflecting, and triceps‐sparing (featured in this article).^26^, ^27^, ^28^ Each comes with its own set of success and trade‐offs.

Recent literature shows that triceps‐sparing (or triceps‐preserving) approaches are associated with faster recovery of elbow extension strength and reduced postoperative morbidity.^27^, ^29^, ^30^ Rates of satisfactory patient‐reported outcomes are also reported to be significantly higher with triceps‐preserving approaches.^29^, ^30^, ^31^, ^32^ However, these techniques may have a steeper learning curve and bone preparation, broaching, and implantation are more difficult when performed through medial and lateral windows. The emphasis on triceps management in TEA is further highlighted by the fact that attempts to address triceps disruption in TEA have documented failure rates ranging from 66% to 100%.^33^ Therefore, as a potential intraoperative modifiable factor to avoid a devastating complication, triceps management is heavily paramount. In complex or revision cases requiring extensive exposure, however, a triceps‐reflecting or splitting approach may be unavoidable.

### Evolving Surgical Indications: Rheumatoid Disease, Osteoarthritis, and Fracture

As the indications for TEA have expanded, efforts to understand how TEA performs in different populations has become an area of interest. Although rheumatoid disease was the classic indication for TEA, profound improvements in detecting and pharmacologically treating rheumatoid disease have significantly helped patients treat their condition^34^ and decreased the number of individuals whose elbow arthritis progress to a stage requiring arthroplasty. Recently, McKissack et al.^35^ used a database study and found that TEA overall decreased in volume from 2010 to 2018 by 37%. TEA for RA experienced the greatest reduction in case volume (58%) and over half (54%) of TEA cases were performed to treat distal humerus fractures or post‐traumatic sequelae.^35^ This aligns with other existing literature that has noted a rise in TEA for complex acute distal humerus fractures.^36^, ^37^, ^38^

Romero et al.^39^ also performed a database study to compare 90‐day and 1‐year outcomes after TEA in 1600 patients who were stratified by surgical indication (RA, osteoarthritis, and fracture). They found that fracture patients had significantly lower rates of triceps injury but higher rates of elbow stiffness. Notably, they found no significant differences in complications between the 3 groups.

Tarallo and colleagues^40^ reported longer‐term results in various patient populations, reporting an overall 85% survival at 10 years and lower survival rates in RA (73%) and osteoarthritis (70%) patients compared with fracture (97%) patients. Implant survival may be better achieved in acute fracture treatment in patients with otherwise adequate bone stock as compared with patients with chronically unstable or eroded elbows as seen in RA. Despite this difference in survival, there were no statistically significant differences in patient‐reported outcomes between the various groups.

It is important to remember that patient indications are not binary—patients may have 2 diagnoses that comprise a more complicated presentation. For example, patients with RA may have lower bone density^41^, ^42^ secondary to treatment medications (i.e., glucocorticoids), making them more susceptible to acute fractures that require TEA. Barco et al.^43^ sought to investigate this and studied 44 cases of TEA performed for comminuted distal humerus fractures with 10‐year follow‐up, reporting 92% survival at 10 years. The authors further stratified the patients into RA versus non‐RA groups, finding that patients with RA who suffered fractures requiring TEA showed a significantly lower survival rate of 76% at 10 years. Although not statistically significant, instances of deep infection, loosening, and periprosthetic fracture were seen more frequently in RA patients. It is possible that the disease‐modifying therapy used in RA populations influences infection rates, osteolysis, and loosening; however, results have been mixed in the existing literature and require further high‐level investigation.^43^, ^44^, ^45^

### Open Reduction Internal Fixation Versus TEA for Distal Humerus Fractures

Distal humerus fractures consist of 2% of all fractures and a third of all humeral fractures.^46^ They occur in a bimodal distribution: young males in high‐energy trauma and elderly osteoporotic females in lower‐energy mechanisms.^47^ ORIF of these fractures is typically employed in younger populations given satisfactory outcomes and avoidance of a lifetime weight‐bearing restriction. However, in elderly patients, the decision between ORIF and TEA remains hotly debated. Several meta‐analyses have reported that reoperation rates are lower with TEA, nonunions are encountered more frequently in ORIF, but overall, patient‐reported outcomes and range of motion do not differ at a clinically relevant level at short‐ and long‐term follow‐up.^40^, ^48^, ^49^, ^50^, ^51^ Given the differing activity restrictions between ORIF and TEA, it is possible that elderly patients accommodate these functional limitations. Investigation into how activity demands evolve postoperatively has yet to be conducted. Overall, treatment of distal humerus fractures depends on a multitude of factors, but it has been suggested that elderly patients with poor bone quality, severe metaphyseal or articular comminution, and significant soft tissue disruption may be better managed with primary TEA.^48^

By synthesizing the peer‐reviewed data and surgical insights discussed in this article, we hope that surgeons are equipped with a nuanced understanding of how to optimize patient outcomes while minimizing complications during TEA.

## DISCLOSURES

The authors (B.K., K.J., A.L.H., J.F.H., X.C.S.) declare that they have no known competing financial interests or personal relationships that could have appeared to influence the work reported in this article.
